# A general auditory bias for handling speaker variability in speech? Evidence in humans and songbirds

**DOI:** 10.3389/fpsyg.2015.01243

**Published:** 2015-08-25

**Authors:** Buddhamas Kriengwatana, Paola Escudero, Anne H. Kerkhoven, Carel ten Cate

**Affiliations:** ^1^Behavioural Biology, Institute for Biology Leiden, Leiden University, Leiden, Netherlands; ^2^Leiden Institute for Brain and Cognition, Leiden University, Leiden, Netherlands; ^3^The MARCS Institute and ARC Centre of Excellence for the Dynamics of Language, University of Western Sydney, Sydney, NSW, Australia

**Keywords:** vowel normalization, zebra finch, vowel categorization, speech perception, comparative cognition

## Abstract

Different speakers produce the same speech sound differently, yet listeners are still able to reliably identify the speech sound. How listeners can adjust their perception to compensate for speaker differences in speech, and whether these compensatory processes are unique only to humans, is still not fully understood. In this study we compare the ability of humans and zebra finches to categorize vowels despite speaker variation in speech in order to test the hypothesis that accommodating speaker and gender differences in isolated vowels can be achieved without prior experience with speaker-related variability. Using a behavioral Go/No-go task and identical stimuli, we compared Australian English adults’ (naïve to Dutch) and zebra finches’ (naïve to human speech) ability to categorize / I/ and /*ε*/ vowels of an novel Dutch speaker after learning to discriminate those vowels from only one other speaker. Experiments 1 and 2 presented vowels of two speakers interspersed or blocked, respectively. Results demonstrate that categorization of vowels is possible without prior exposure to speaker-related variability in speech for zebra finches, and in non-native vowel categories for humans. Therefore, this study is the first to provide evidence for what might be a species-shared auditory bias that may supersede speaker-related information during vowel categorization. It additionally provides behavioral evidence contradicting a prior hypothesis that accommodation of speaker differences is achieved via the use of formant ratios. Therefore, investigations of alternative accounts of vowel normalization that incorporate the possibility of an auditory bias for disregarding inter-speaker variability are warranted.

## Introduction

It is remarkable that even though different speakers produce the same speech sound differently, listeners are still able to identify the speech sound. How humans are able to seemingly effortlessly adjust their perception to accommodate for speaker differences in speech has long been a topic of great scientific interest. The reason for this is because speech perception is thought to involve categorization and mapping of a highly variable acoustic signal onto linguistic representations (Holt and Lotto, 2010). A compelling example of our ability to categorize speech sounds despite enormous variability arising from speaker differences is in the case of vowels. Vowels can be characterized by specific frequencies called formant frequencies, which are produced when the position of the jaw, lips, and tongue modulate the acoustic resonances of the vocal tract. The acoustic analysis of vowels usually involves measuring its first and second formant frequencies (F1 and F2) using the acoustic Hertz scale (Colatoni et al., 2015a). The same vowels spoken by men, women, and children differ markedly in F1 and F2, causing large variation within a vowel category and striking overlap between vowel categories (Potter and Steinberg, 1950; Peterson and Barney, 1952; Hillenbrand et al., 1995). These differences originate from morphological differences in vocal folds, vocal tract size and length, with men tending to produce lower formants than women and children due to their longer vocal tracts (Fitch and Giedd, 1999). Despite these differences, human adults (Peterson and Barney, 1952; Strange et al., 1976; Assmann et al., 1982), pre-linguistic infants (Kuhl, 1983), and even non-human animals (Dewson, 1964; Dewson et al., 1969; Baru, 1975; Burdick and Miller, 1975; Dooling and Brown, 1990; Ohms et al., 2010a) can effectively categorize vowels of different speakers and genders. How are human adults, infants, and non-human animals able to compensate for speaker differences in vowel production, and are they all doing it in the same way?

One method of dealing perceptually with speaker variability in vowel production is to eliminate speaker variability by transforming the speech signal in a way that minimizes speaker differences within a vowel category while maximizing differences between vowel categories. Various methods have been proposed (Adank et al., 2004a; Escudero and Bion, 2007; Johnson, 2008), including a class of formant ratio hypotheses. These hypotheses suggest that vowels are perceived, not in terms of raw formant frequencies such as F1 and F2, but instead are perceived in terms of formant frequency ratios (such as the second formant frequency divided by the third formant frequency, F2/F3; Potter and Steinberg, 1950; Syrdal and Gopal, 1986; Miller, 1989; Monahan and Idsardi, 2010). There is neuropsychological evidence to support the idea that vowel perception may occur at the level of formant ratios, and that perception of formant ratios occurs at low levels of auditory processing. For instance, the human brain is sensitive to the F1/F3 ratio (Monahan and Idsardi, 2010), and extraction and processing of vowel formants appears to be pre-attentive and occurs at the primary auditory cortex or subcortical level (von Kriegstein et al., 2006; Edmonds et al., 2010; Tuomainen et al., 2013). Moreover, transformations of formant frequency values into formant ratios effectively eliminate speaker and gender differences in a corpus of American English vowels (Monahan and Idsardi, 2010). Therefore, perceiving vowels in terms of formant ratios could explain how humans are able identify vowels and accommodate for the enormous signal variability emanating from speaker and gender differences in vowel production.

Perceptual adjustments to accommodate for speaker differences in vowels via perception of formant ratios that requires only pre-attentive and low level processing mechanisms may explain why even very young infants can accommodate for speaker differences in vowel production (Kuhl, 1983). Combined with findings suggesting non-human animals also adjust for speaker differences (e.g., Burdick and Miller, 1975; Dooling and Brown, 1990; Ohms et al., 2010a), this raises an intriguing possibility that accommodation for speaker differences in vowel production may arise from the tendency of the vertebrate auditory system to perceive formant ratios. If this is the case, then exposure to speaker-variability in vowel production need not be necessary in order for listeners to compensate for speaker and gender differences.

However, the currently available animal studies do not allow a firm conclusion on this issue for several reasons (Kriengwatana et al., 2015). First, they used /i/, /u/, and /a/ vowels as stimuli (Dewson, 1964; Dewson et al., 1969; Baru, 1975; Burdick and Miller, 1975). As these vowels occupy distant acoustic spaces, the variability between these vowel categories is likely to be larger than the variability between speakers. Consequently, a stronger test for vowel normalization would be to use vowel categories where speaker differences and vowel category differences have relatively equal variation. A second factor preventing firm conclusions is that some studies did not test the ability to normalize vowels across genders (Dooling and Brown, 1990), even though much of the acoustic overlap between vowel categories is due to gender differences (Peterson and Barney, 1952). Finally, the use of synthetic vowels that differ in only a single acoustic parameter, e.g., using synthesized vowels with only variation in fundamental frequency (Eriksson and Villa, 2006; Bizley et al., 2013), yields unconvincing evidence for the ability to adjust to different speakers because voices differ in various dimensions, and no single acoustic cue has been found that reliably predicts speaker characteristics (Creel and Bregman, 2011).

In a study that used natural stimuli from male and female speakers (Ohms et al., 2010a), zebra finches could generalize their discrimination of two words that differ only in vowels (w?t and w*ε*t). Discrimination was found after exposure to a single speaker and then multiple speakers of the one sex, which generalized to novel speakers of the other sex. Therefore, this study shows that non-human animals can generalize their discrimination of vowels from female speakers to male speakers and vice versa, if they have had prior experience with discriminating vowels from speakers of the other sex. However, for this study as well as for previous ones on both humans and non-human animals, it is not clear whether experience with many different speakers is required for successful normalization of speaker and gender variability in vowel production.

Certainly, what is lacking is an experiment that (1) tests for compensation of speaker differences if variability between vowel categories is as large as variability within vowel categories; (2) tests whether subjects can normalize vowels of different males and female speakers without having been previously trained with many speakers (i.e., exposed to between-speaker variability in vowel production); and (3) uses isolated vowels rather than words as used by Ohms et al. (2010a) to provide a stronger test of the ability to accommodate speaker differences in vowel production, as isolated vowels are much shorter in duration and consequently contain much less information than words (where consonants may also have an additional influence).

The present study attempts to fill this gap by testing whether normalization of speaker and gender differences in isolated vowels requires prior experience with speaker-related variability. Here, we define normalization as perceptual transformations of acoustic input to allow behavioral classification of vowels into functionally equivalent categories. Using a comparative approach, we test this hypothesis in two species: songbirds that are naïve to human speech and human adults that are naïve to the Dutch stimuli chosen for this study. We compare humans and songbirds for two reasons. First, tests in songbirds will determine whether the tendency for the auditory system to normalize speaker and gender differences in vowels (even without experience with different speakers) is shared with other species and therefore might have been a pre-existing, or at least, an independently evolved property that has affected speech evolution. Second, tests in adult Australian English speakers will determine whether experience with speaker and gender variability of a particular vowel category is required for successful normalization. In our study we use the zebra finch as a songbird model. The zebra finch is used worldwide as a model species to study many aspects of song development and its neurobiological and molecular basis (e.g., Haesler et al., 2007; Mooney, 2009). It is also a comparative model to study whether and how songbirds can process different aspects of human speech sounds (e.g., Ohms et al., 2010a, 2012; Spierings and ten Cate, 2014) and artificial grammar patterns (e.g., van Heijningen et al., 2009; Chen et al., 2015).

**FIGURE 1:**
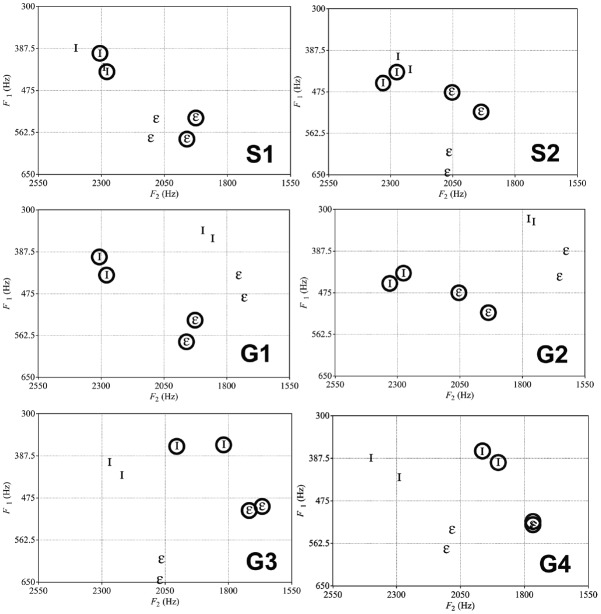
**F1 and F2 values for the sets of speakers used for speaker and gender normalization conditions.** S1 and S2 show the two stimuli sets used for Speaker normalization (all female tokens). Circled tokens were the *FAM* Go and No-go tokens used during the training, non-circled ones were the *NEW* test tokens. G1, G2, G3, and G4 were the stimulus sets used for Gender normalization (male and female tokens). In sets G1 and G2, the *FAM* Go and No-go tokens (circled) were female and the *NEW* test tokens male. In sets G3 and G3, the *FAM* Go and No-go tokens were male and *NEW* tokens female. In Experiment 1, birds were exposed to one of the stimulus sets, while humans were exposed to an S stimuli set and a G stimuli set (e.g., S1 and G1, G2 and S1). In Experiment 2, birds and returning human participants were exposed to the same set as Experiment 1.

In two experiments, humans and zebra finches were tested with the Go/No-go task using identical stimuli: both species learned to discriminate the Dutch vowels / I/ and /*ε*/ from a single speaker (i.e., familiar Dutch speaker, *FAM*) and were tested on their ability to discriminate the same vowels produced by a different speaker (i.e., novel Dutch speaker of the same sex or different sex, *NEW*; Figure 1). In Experiment 1 *FAM* and *NEW* tokens were presented in a mixed-speaker design and in Experiment 2 *FAM* and *NEW* tokens were presented in blocked-speaker design; both experiments used the same stimuli. The Go/No-go task requires subjects to make a response toward vowel stimuli assigned to one category (Go) and to inhibit responses toward vowel stimuli assigned to the other category (No-go). If experience with speaker variability in vowel production is necessary for successful normalization to occur, then we predicted that zebra finches and humans would not be able to discriminate the vowels of the second, new speaker.

## Experiment 1: Mixed-Speaker Design

### Subjects

Twelve adult zebra finches (five males, seven females) were tested on the speaker normalization and another 12 adult zebra finches (six males, six females) were tested on the gender normalization (for explanations of speaker and gender normalization conditions, refer to Stimuli section and Figure 1). All subjects were from the Leiden University breeding colony and were at least 120 days old at the start of the experiment. Prior to experiments, subjects were housed in same-sex aviaries and maintained on a 13.5 L: 10.5 D schedule at 20–22°C with food (commercial tropical seed mixture), grit, cuttlebone and water available *ad libitum*.

Forty undergraduate students (29 females, mean age = 21.9 years, SD = 6.41; 29 multilinguals) at the University of Western Sydney participated in the experiments in exchange for course credit. Data for this study were part of a larger dataset collected for Kriengwatana et al. (unpublished). Some listeners were monolingual Australian English, while others spoke languages in addition to English (Arabic, French, Chinese, Serbian, Korean, Vietnamese, Iranian, Mandarin, Khmer, Indonesian, Hindi, Vietnamese, Italian, Urdu, Assyrian, German, Greek, Bengali, Egyptian, Macedonian, Cantonese, Tagalog, Polish, Portuguese). None of the listeners had prior exposure to Dutch. Each testing session lasted 1 h, with each experiment taking 25 min to complete. Each participant completed both speaker and gender normalization conditions (order counterbalanced; see Figure 1).

### Stimuli

Stimuli were naturally produced Dutch vowels / I/ and /*ε*/ extracted from a [s-Vowel-s] context by male and female speakers from Adank et al. (2004b). Two tokens of each vowel from each speaker were used. We ramped and resampled all tokens to 44100 Hz and equalized duration and amplitude using PRAAT software (Boersma, 2001). We included tokens from several different speakers to ensure that the results we obtained were generalizable (Figure 1). Whether the / I/ or /*ε*/ vowel was the Go or No-go stimuli was counterbalanced across birds and human participants.

For speaker normalization, there were two pairs of female speakers (Figure 1), counterbalanced for which vowel category was the assigned as the Go or No-go stimulus in the Go/No-go task. For gender normalization there were four pairs of male and female speakers, also counterbalanced as in speaker normalization (Figure 1).

### Apparatus

Birds were tested individually in an operant conditioning cage (70 cm × 30 cm × 45 cm LWH) in a sound attenuated room. The front and sides of the cage were made from wire mesh and the back panel of the cage was made of plywood with access to two red LED sensors and a food hatch. A speaker located 1 m above the cage played the stimuli at 70 dB. A fluorescent bulb was on the top of the cage maintained the same light–dark schedule as the breeding colony, and was switched off when birds received a penalty for incorrect responses during the Go/No-go task. Water, cuttlebone, and grit were available *ad libitum*. All animal procedures were approved by the Leiden University animal experimentation committee (DEC #13151).

Humans were presented stimuli via headphones attached to a laptop computer running E-prime version 2 in a quiet location.

### Go/No-Go Procedure

Birds could initiate a trial by pecking the illuminated left LED sensor, which led to the presentation of a vowel stimulus, and illumination of the right LED sensor. Birds were rewarded with 10 s of access to food if they pecked the right sensor within 6 s of hearing a Go stimulus, and were penalized with a 15 s period of darkness for pecking the right sensor within 6 s of hearing a No-go stimulus. Each trial was separated by a 2 s interval where both LED sensors were extinguished and unresponsive to pecks.

Birds were pre-trained with a recording of a zebra finch song as the Go and a 2 k-Hz pure tone as the No-go stimulus until they reached a learning criterion of at least 75% responses to the Go and less than 25% responses to the No-go stimulus for two consecutive days. Birds were then switched to the training phase, where they learned to discriminate between *FAM* / I/ and /*ε*/ until they reached the learning criterion of at least 75% responses to the Go and less than 25% responses to the No-go stimuli for three consecutive days. Once this criterion was reached, birds proceeded to the testing phase, where reward and penalties were provided on only 80% of trials—in this 80% of trials only *FAM* tokens were presented. In the remaining 20% of trials, the / I/ and /*ε*/ tokens from *NEW* were presented interspersed with *FAM* tokens. For some birds *NEW* tokens were from a speaker with the same sex as *FAM*; for others *NEW* tokens were from a speaker with a different sex as *FAM*. No feedback was given for this 20% of trials to ensure that subjects’ responses to the unfamiliar speaker did not reflect learning due to feedback. We collected 40 responses to each token of *FAM* and *NEW* (80 responses per vowel type per speaker). We adapted the zebra finch Go/No-go task for use with human adult participants so that we could directly compare the performance of birds and humans. Listeners were given minimal verbal instructions, and were simply told to listen to sounds and to determine what to do in order to earn as many points as possible. To earn points, participants had to press “spacebar” when they heard the Go stimulus, and to not press any key when they heard the No-go stimulus.

Participants initiated each trial by pressing the spacebar. After the presentation of a token, the text “Press Spacebar if appropriate” appeared on the screen. If participants pressed the spacebar within 2 s after hearing a Go stimulus, they were positively reinforced with a “smiley face,” a pleasant sound, and 1 point. If they pressed the spacebar within 2 s after hearing a No-go stimulus, they were penalized with a “sad face,” an unpleasant sound, and no point. Feedback appeared on the screen for 2 s.

Similar to birds, human participants completed a pre-training phase (two blocks of 10 randomized trials of “deet” and “pon”), a training phase (three blocks of 20 randomized trials of *FAM* / I/ and /*ε*/, total 60 trials), and a test phase (six blocks of 20 randomized trials of *FAM* and *NEW*, total 120 trials with 96 *FAM* and 24 *NEW* tokens). Participants were not informed that feedback would not be given for responses to unfamiliar vowel tokens or to 25% of responses to trained tokens. In this way, we obtained six responses to each token of the familiar and unfamiliar speaker (12 responses per vowel type per speaker) in the absence of feedback. We only collected 12 responses to *NEW* for humans in Experiment 1 in order to prevent fatigue. Although this differs from the number of responses we collected from the zebra finches, it is unlikely to affect our comparison because humans also learned the Go/No-go task and *FAM* discrimination faster than the zebra finches.

### Statistics

We used generalized linear mixed models (GLMM) with a binary logistic regression and the number of Go responses to each vowel token from *FAM* and *NEW* on trials without feedback as the dependent variable. We first analyzed for effects of stimulus set (Figure 1) on number of Go responses in a separate GLMMs for birds and humans and found no significant differences between speaker sets, so we pooled results from the different speaker sets in the final model.

For birds, the final model contained normalization type (speaker or gender), stimulus type, and normalization type × stimulus type as fixed effects, with stimulus type specified as a within-subject variable. For humans, the final GLMM model contained normalization type, stimulus type, linguistic background (monolingual or multilingual), and order of testing (speaker or gender normalization first) as fixed main effects. We entered all two- and three-way interactions between stimulus type and other terms as fixed interaction effects. Stimulus type was specified as a within-subject variable for birds. Stimulus type and normalization type were specified as within-subject variables for humans. Satterthwaite correction for degrees of freedom was applied, as required when performing GLMMs that use pseudolikelihood estimation (Stroup, 2012).

### Results and Discussion

**FIGURE 2:**
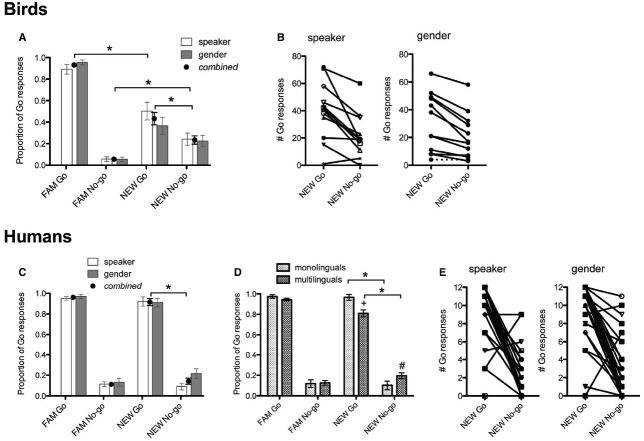
**Group level performance of birds (A) and humans (C), and individual level performance of birds (B) and humans (E) in Experiment 1.** Dotted line in **(B)** shows performance of bird 257 (discussed in Experiment 2 Results). In **(A,C)**, black dots represent the combined average of performance on speaker and gender normalization conditions (which were not significantly different from each other). Birds and humans correctly discriminated between *NEW* vowels because they responded significantly more to the Go than No-go tokens (*). **(D)** Multilinguals’ responses to the *FAM* Go and *NEW* Go differed significantly (+), as did their responses to the *FAM* No-go and *NEW* No-go (#). Nevertheless, monolingual and multilinguals correctly discriminated between the *NEW* Go and *NEW* No-go (*).

Our analysis of birds’ performance showed that they could discriminate between the *NEW* / I/ and /*ε*/ tokens, whether they originated from the same or different gender as *FAM* (Figure 2A). There was a significant main effect of stimulus type (*FAM* Go, *FAM* No-go, *NEW* Go, *NEW* No-go) on number of Go responses [*F*(3,19) = 80.0, *p* < 0.001], but no main effect of experiment (speaker or gender normalization) or interaction of stimulus type by experiment. To assess differences between responses to stimulus types, three simple planned comparisons were performed between: (1) *FAM* Go and *NEW* Go; (2) *FAM* No-go and *NEW* No-go; (3) *NEW* Go and *NEW* No-go. All three comparisons differed significantly from each other (*p* < 0.001). This indicates that birds noticed the speaker/gender change, but still differentiated vowel categories by responding more to the unfamiliar Go stimuli than unfamiliar No-go stimuli (Figure 2A). There were noticeable individual differences in performance, as some birds strongly and correctly discriminated between the vowels of the unfamiliar speaker while other birds did not or discriminated incorrectly (Figure 2B).

The analysis of human’s performance showed a partly similar pattern to birds. The number of Go responses differed significantly depending on stimulus type [main effect stimulus type, *F*(3,156) = 74.6, *p* < 0.001], but did not differ by normalization type, linguistic background, or order of testing (all humans did a speaker and gender normalization experiment). This indicates that participants responded similarly regardless of whether they were doing speaker or gender normalization (Figures 2C,E), and that performance on the second normalization task was not affected by experience with the first normalization task. In contrast to the findings in the zebra finches, simple planned comparisons of the main effect showed that only the *NEW* Go and *NEW* No-go differed significantly (*p* = 0.019). There was a significant interaction of stimulus type × linguistic background (*p* = 0.044; Figure 2D). *Post hoc* comparisons with Bonferroni adjustment conducted separately for monolinguals and multilinguals showed that multilinguals did not categorize the *NEW* vowels as strongly as monolinguals, in speaker and gender normalization experiments. Multilinguals responded differently to all stimulus types (*p* < 0.001 for all comparisons except *FAM* No-go and *NEW* No-go, where there was a trend to respond more to the *NEW* No-go, *p* = 0.051), whereas monolinguals did not respond differently to *FAM* Go and *NEW* Go, or to *FAM* No-go and *NEW* No-go (Figure 2D). Hence, multilingual human listeners performed more similarly to zebra finches than monolingual human listeners. Humans also showed noticeable individual differences in their classification of *NEW* vowels, similar to the birds (Figure 2E). These results show that despite being influenced by speaker/gender changes (albeit to a lesser degree than birds), human participants can correctly classify non-native vowels of an unfamiliar speaker. Nevertheless, human vowel normalization can be influenced by linguistic experience (with knowledge of more than one language reducing discrimination).

Therefore, the results of Experiment 1 show that most humans and at least some birds can correctly classify novel or non-native vowels of produced by a different speaker, regardless of gender, without prior exposure to speaker/gender-related variability in production of these vowels. Nevertheless, due to differences in experimental design, the current zebra finch findings are not directly comparable with several prior studies on vowel normalization that exposed animals to blocks of different speakers (Dewson, 1964; Dewson et al., 1969; Baru, 1975; Burdick and Miller, 1975; Ohms et al., 2010a). The mixed-speaker design used in Experiment 1 presented vowels of the *FAM* and *NEW* simultaneously in the testing phase (i.e., speakers are mixed, not blocked). Mixed-speaker designs have also been used in other experiments that have tested speech perception non-human animals (e.g., Dooling and Brown, 1990; Ohms et al., 2012). Researchers have so far assumed that these different designs measure the same cognitive processes, but this assumption has yet to be explicitly validated. Consequently, we conducted another experiment with a blocked-speaker design to test and compare vowel normalization abilities of zebra finches and humans. We predicted that both species would still be able to normalize speaker differences in vowel production if speakers are presented sequentially, as prior work in humans suggests that vowel identification is easier in blocked-speaker conditions compared to mixed-speaker conditions (Strange et al., 1976; Assmann et al., 1982; Magnuson and Nusbaum, 2007).

## Experiment 2: Blocked-Speaker Design

### Subjects

The same birds that completed Experiment 1 were tested in Experiment 2. In Experiment 2, birds heard the same set of speakers as in Experiment 1. After completing Experiment 1, birds were given a rest period of 7–15 days (*ad libitum* access to food, water, grit, and cuttlebone) before being starting Experiment 2.

Twenty-five undergraduate students at the University of Western Sydney participated in the experiments in exchange for course credit. Ten participants had previously participated in Experiment 1 (nine females, mean age = 23.0 years, SD = 8.17; 11 multilinguals); 15 were naïve and had not (11 females, mean age = 20.6 years, SD = 2.77; 10 multilinguals). Four of the naïve participants completed the gender normalization with a different set of speakers than the ones used in the present study, so only their data for speaker normalization was included. Similar to Experiment 1, participants had diverse language backgrounds and many spoke other languages in addition to English. None of the listeners had prior exposure to Dutch.

### Stimuli, Apparatus, and Procedure

The same stimuli and apparatus used in Experiment 1 were used in Experiment 2. The same Go/No-go procedure was also used, where humans and birds had to learn to respond to one set of stimuli and withhold responses to another set of stimuli.

Zebra finches started Experiment 2 by repeating the pre-training phase and training phase with the same Go/No-go task, apparatus, and stimuli as in Experiment 1. When the learning criterion in the training phase was reached (at least 75% correct for three consecutive days), birds completed a second training phase (with the same learning criterion), where the *FAM* tokens were replaced by *NEW* tokens. Therefore, Experiment 2 blocked-speaker design presents vowels of *FAM* and *NEW* in separate blocks with trial-by-trial feedback for the tokens of both speakers. Humans were tested using the same Go/No-go task, apparatus, and stimuli as Experiment 1. Participants completed a pre-training phase (20 trials of “deet” and “pon”) and a training phase (60 trials *FAM* / I/ and /*ε*/). They then completed a second training phase (with feedback) consisting of three blocks of 20 randomized trials of *NEW* / I/ and /*ε*/. The trials immediately after the switch from *FAM* to *NEW* are critical for determining whether subjects can generalize their discrimination of *FAM* / I/ and /*ε*/ to *NEW* / I/ and /*ε*/ tokens.

### Statistics

We used a GLMM with a binary logistic regression and the number of Go responses to *FAM* and *NEW* tokens as the dependent variable. For birds, this was the number of Go responses toward *FAM* tokens on the day before the switch to *NEW* tokens, and the first 40 presentations of each of the *NEW* vowel tokens (immediately after the switch from *FAM* to *NEW*). Normalization type, stimulus type, and the interaction of normalization type and stimulus type were entered as fixed effects. Stimulus type was also specified as a within-subject variable. For humans, the dependent variable was the last 30 presentations of *FAM* tokens and the first 30 presentations of each *NEW* token, respectively. Normalization type, stimulus type, status (returning or new participants), linguistic background, and all higher-order interactions including stimulus type were entered as fixed effects (except for the three-way interaction involving stimulus type, status, and linguistic background because none of our returning subjects were monolingual). Normalization type and stimulus type were specified as within-subject variables. We did not include order of testing as a factor since it did not seem to influence participants’ performance in Experiment 1. For birds and humans we again found no differences between responses to the different speaker sets, so we pooled results from the different speaker sets in the final model. Satterthwaite correction for degrees of freedom was applied.

### Results and Discussion

**FIGURE 3:**
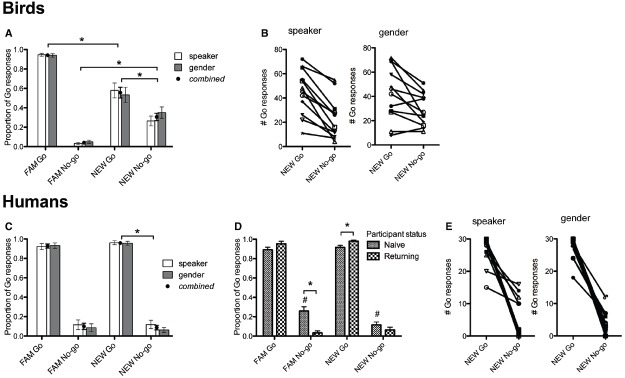
**Group level performance of birds (A) and humans (C), and individual level performance of birds (B) and humans (E) on Experiment 2.** Black circles represent the combined average of performance on speaker and gender normalization conditions (which were not significantly different from each other). Asterisks in **(A)** and **(C)** indicate that birds and humans correctly discriminated between *NEW* vowels because they responded significantly more to the Go than No-go tokens. Naïve participants performed worse than returning participants **(D)** in categorizing *FAM* No-go and *NEW* Go (*), but seemed to improve in their ability to categorize No-go tokens over trials (#). Note that for birds and multilinguals, *FAM* Go is significantly different from *NEW* Go (not indicated in the figure).

As in Experiment 1, humans and birds were tested on their categorization of *NEW* vowels after having first learned to discriminate *FAM* vowels. In line with our findings in Experiment 1, birds and humans could discriminate between the *NEW* / I/ and /*ε*/ tokens in a blocked-speaker design, regardless whether *NEW* was of the same or different gender (Figure 3). For birds, there was a significant main effect of stimulus type on number of Go responses, *F*(3,36) = 236.8, *p* < 0.001. Simple planned comparisons showed that *FAM* Go and *NEW* Go, *FAM* No-go and *NEW* No-go, and *NEW* Go and *NEW* No-go differed significantly from each other (*p* < 0.001). This indicates that, as in Experiment 1, birds were sensitive to the speaker/gender change, but maintained their distinction of vowel categories (Figure 3A). No other main effects or interactions were significant. For humans, there was a significant main effect of stimulus type [*F*(3,79) = 81.9, *p* < 0.001] and interaction between stimulus type and status [*F*(3,79) = 7.2, *p* < 0.001]. Simple planned comparisons of the main effect showed that humans classified *NEW* tokens similarly to *FAM* tokens, while distinguishing between *NEW* Go *NEW* No-go tokens (*p* < 0.001; Figure 3C). Participants who had done Experiment 1 performed better than naïve participants when classifying *FAM* No-go (*p* < 0.001) and *NEW* Go tokens (*p* = 0.007, Bonferroni-corrected; Figure 3D). Naïve participants also differed in categorization of FAM No-go and NEW No-go, showing better categorization with NEW tokens (*p* < 0.001; Figure 3D). There were again noticeable individual differences in performance: some birds and humans robustly discriminated between *NEW* vowels and others did not or discriminated incorrectly (Figures 3B,E). Therefore, these results show that the ability to normalize speaker and gender differences in vowels is robust despite differences in experimental design (i.e., Experiment 1 was a mixed-speaker and Experiment 2 was a blocked-speaker design). Our findings are thus comparable to previous studies that have used either one or the other type of design and additionally support the assumption that mixed- and blocked-speaker designs measure the same cognitive processes.

The results of both Experiments 1 and 2 suggest that birds and humans can successfully normalize speaker and gender differences in vowel production without prior exposure to speaker-related variability (in the case of birds), or without exposure to speaker-related variability in specific (non-native) vowel categories (in the case of humans). Nevertheless, when looking at the speaker sets used in this study (Figure 1), one may question whether normalization (i.e., executing a perceptual transformation of the auditory input) was truly necessary for birds or humans to correctly categorize *NEW* vowels used in the present study. In many cases the acoustic distance between the F1 and/or F2 formant frequencies of the same vowel categories of the *FAM* and *NEW* are smaller than the acoustic distance in F1 and/or F2 formant frequencies between vowel categories (e.g., Figure 1, stimulus set S1). Thus, our analyses so far have not excluded that birds’ or humans’ successful performance was simply due to reliance on acoustic distances and hence could be explained by generalization of a single parameter from the *FAM* to the *NEW*, rather than by normalization.

## Can acoustic Distance or Formant Ratios Explain Successful Categorization Performance?

**FIGURE 4:**
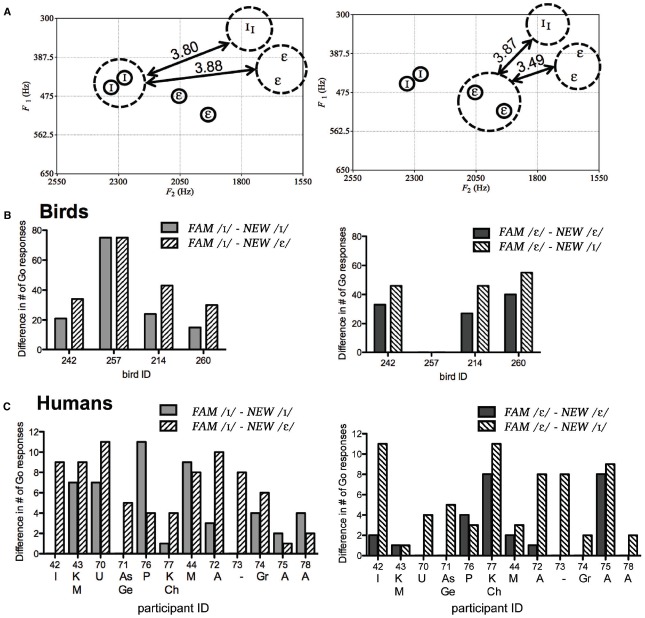
**Plots of acoustic distances between *FAM* (circled) and *NEW* vowels in the G2 stimulus set of the gender normalization experiment (A) show that acoustic distances within and between vowel categories of familiar and unfamiliar speaker are highly similar and thus likely an unreliable cue for category membership.** Still, birds **(B)** and humans **(C)** show differences in response to *NEW* vowels when the stimulus is the same (solid bars) or a different vowel (striped bars). Smaller values indicate smaller perceived differences. Bird 257 treated all *NEW* stimuli as No-go stimuli. Letters underneath human participant numbers indicate languages spoken other than English (I, Iranian; K, Khmer; M, Mandarin; U, Urdu; As, Assyrian; Ge, German; P, Persian; Ch, Chinese; A, Arabic; Gr, Greek).

To determine whether acoustic distances explains successful categorization of *NEW* tokens by humans and birds, we focused our subsequent analysis on birds’ and humans’ responses to a specific speaker set where the acoustic distances within and between vowel categories of the two speakers were more similar (Figure 1, stimulus set G2). For this speaker set, we calculated the acoustic distances between: (1) *FAM* / I/ and *NEW* / I/; (2) *FAM* /*ε*/ and *NEW* /*ε*/; (3) *FAM* / I/ and *NEW* /*ε*/; (4) *FAM* /*ε*/ and *NEW* / I/ (Figure 4). Thus, the first two calculations represent within vowel category differences and the latter two calculations represent between vowel category differences. Acoustic distances were calculated as √[F1^2^ + F2^2^ + F3^2^] in Erb. We also calculated acoustic distances that included F0 (in Mel) as an index of speaker quality, using the formula F0 × √[F1^2 ^+ F2^2^ + F3^2^], which yielded similar between- and within-category differences as calculations with formants only (*FAM* / I/ to *NEW* / I/ = 49.65; *FAM* /*ε*/ to *NEW* /*ε*/ = 36.17; *FAM* / I/ to *NEW* /*ε*/ = 47.16; *FAM* /*ε*/ to *NEW* / I/ = 38.69).

These calculations show that the acoustic distances between and within vowel categories (i.e., acoustic distance of the *NEW* vowels to each of the *FAM* vowels) are highly similar. Therefore, acoustic distance is an uninformative cue with which to categorize the *NEW* vowels. Using data from Experiment 1 (where no feedback was provided so results were not biased by learning), we computed the perceived similarity between *FAM* and *NEW* vowels by taking the absolute difference between Go responses to *FAM* and *NEW* vowels (i.e., *FAM* / I/ and *NEW* / I/; *FAM* /*ε*/ and *NEW* /*ε*/; *FAM* / I/ and *NEW* /*ε*/; *FAM* /*ε*/ and *NEW* / I/). Again, the first two calculations represent the perceived similarity of vowels within the same category (within-category) and the latter two calculations represent the perceived similarity of vowels of different categories (between-category). If an individual was relying on acoustic distances, then responses to within- and between-categories should be relatively equal since the acoustic distances within- and between-categories are quite similar.

However, most of the birds and human listeners did not respond equally to / I/ and /*ε*/ vowels (Figure 4). Paired *t*-tests of birds’ responses showed that birds treated *NEW* / I/ almost significantly more like *FAM* / I/ than *FAM* /*ε*/ (*p* = 0.065). Birds also treated *NEW* /*ε*/ as more almost significantly more similar to *FAM* /*ε*/ than *FAM* / I/ (*p* = 0.065). However, bird 257 behaved very differently from the others by treating all *NEW* tokens as No-go stimuli, suggesting that it had learned to discriminate between *FAM* vowel categories by simply memorizing the tokens (see Figure 2B). Paired *t*-tests excluding bird 257 showed that birds treated *NEW* / I/ as significantly more similar to *FAM* / I/ (mean ± SEM = 20.0 ± 2.65) than *FAM* /*ε*/ (mean ± SEM = 35.67 ± 3.74), *t*(2) = –8.88, *p* = 0.012, and treated *NEW* /*ε*/ as significantly more similar to *FAM* /*ε*/ (mean ± SEM = 33.33 ± 3.76) than *FAM* / I/ (mean ± SEM = 49.00 ± 3.00), *t*(2) = –8.88, *p* = 0.012. Analysis of human data showed that human participants did not treat *NEW* / I/ as more similar to *FAM* / I/ (mean ± SEM = 4.00 ± 1.08) than *FAM* /*ε*/ (mean ± SEM = 6.42 ± 0.93; *P* = 0.099), although they did treat *NEW* /*ε*/ as significantly more similar to *FAM* /*ε*/ (mean ± SEM = 2.17 ± 0.86) than *FAM* / I/ (mean ± SEM = 5.58 ± 1.05), *t*(11) = –3.66, *p* = 0.004. Thus, neither birds nor humans appear to be consistently using acoustic distances for vowel identification, at least for this particular speaker set.

**FIGURE 5:**
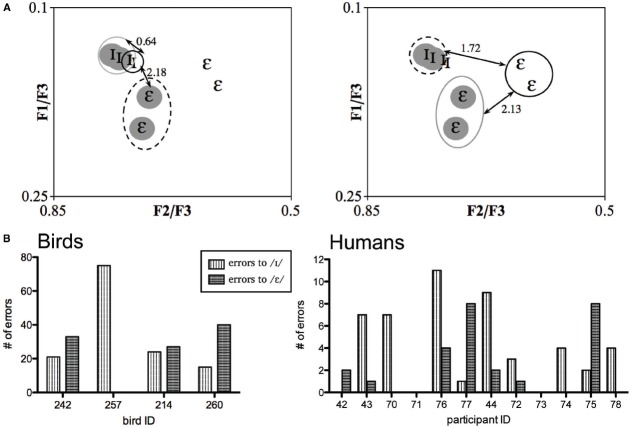
**Acoustic distances between vowel categories of ***FAM*** and ***NEW*** (gray) after transformation into formant ratios according to Monahan and Idsardi (2010; A), and the number of errors individuals tested on this speaker set made when classifying *NEW* / I/ and /*ε*/ (B)**.

Recently, Monahan and Idsardi (2010) demonstrated that transformations of first and second formant frequency values of English vowels (4) into F1/F3 and F2/F3 ratios effectively eliminated variation between speakers and genders (where F1, F2, and F3 are the first, second, and third formant frequencies, respectively). They suggested that F3 varies correlational with a speaker’s fundamental frequency and remains consistent across vowels for that speaker (2,21). Therefore, we subsequently analyzed whether transformation of formant frequencies into F1/F3 and F2/F3 formant ratios (Monahan and Idsardi, 2010), could explain how birds and humans might have behaviorally classified vowels in this speaker set. We observed that after transformation into ratios, the / I/ tokens of *FAM* and *NEW* were tightly clustered whereas the /*ε*/ tokens were not (Figure 5). Computations of acoustic distances within and between vowel categories in this transformed space using the formula √[(*NEW* F1/F3 – *FAM* F1/F3)^2^ + (*NEW* F2/F3 – *FAM* F2/F3)^2^] supports this observation: the *NEW* / I/ vowels are acoustically much closer to the *FAM* / I/ than /*ε*/ vowels, but the *NEW* /*ε*/ vowel are acoustically almost equidistant between the *FAM* / I/ and /*ε*/ vowels (Figure 5). Consequently, if individuals used formant ratios to classify the *NEW* vowels, then both birds and humans should be significantly more accurate at categorizing the *NEW* / I/ vowels than /*ε*/ vowels.

To determine accuracy we again used data from Experiment 1 and calculated error rates for each individual, which we defined as the absolute difference between the number of responses made to *NEW* minus the number of responses made to the *FAM* during the test phase. For example, bird 242 received / I/ as the Go stimulus and /*ε*/ as the No-go stimulus, and made Go responses to the *FAM* and *NEW* / I/ and /*ε*/ vowels 73 and 52, and 6 and 39 times, respectively. Thus, this bird made 21 (73 – 52 = 21) and 33 (6 – 39 = 33) errors when classifying the / I/ and /*ε*/ categories, respectively. Results from all individuals tested in this speaker set are shown in Figure 5. We found that three out of four birds and three out of twelve human listeners made more errors when categorizing the *NEW* /*ε*/ than / I/. Paired *t*-tests on error rates showed that neither birds nor humans made significantly more errors to /*ε*/ than / I/ (mean ± SEM for errors to /*ε*/ and / I/ = 25.00 ± 8.75 and 33.75 ± 13.88 for birds; 2.17 ± 0.86 and 4.00 ± 1.08 for humans). The analyses include bird 257, as removal of its data did not alter results. Thus, our behavioral data do not support the hypothesis that sensitivity to formant ratios accounts for how humans normalize vowels, at least not at the overt behavioral level (Monahan and Idsardi, 2010). Nevertheless, our null results may have resulted from low statistical power (η^2^ = 0.048 and 0.131 for birds and humans, respectively) and differences in linguistic background, so further experiments with larger sample sizes and a homogenous linguistic participant population are warranted.

## General Discussion

Our findings in songbirds and human adults clearly show that categorization of isolated vowels irrespective of speaker and gender differences does not require prior experience with speaker-related variability. Specifically, we have shown that at the group level, zebra finches can, like humans, correctly categorize vowels of an unfamiliar speaker if they previously learned to discriminate the vowels of another speaker (note that inter-individual variability in performance was displayed by both zebra finches and humans). We demonstrate this with two different experimental designs (i.e., mixed- and blocked-speaker designs) that have been used by many prior studies to test vowel discrimination/generalization. Importantly, these results were obtained with stimuli chosen to overcome problems in previous animal studies by ensuring that (1) the variability between vowel categories was similar to the variability between speakers; (2) natural vowel tokens were used; (3) multiple sets of speakers were used; (4) stimuli included male and female speakers. Prior studies did not use stimuli or designs that encompassed all these aspects (Dewson, 1964; Dewson et al., 1969; Baru, 1975; Burdick and Miller, 1975; Dooling and Brown, 1990; Eriksson and Villa, 2006; Bizley et al., 2013; Kriengwatana et al., 2015). The use of isolated vowels also eliminates the possibility that the birds could have relied on other information, such as formant transitions in words, to successfully categorize vowels (Ohms et al., 2010a). Consequently, our study provides the strongest evidence thus far that non-human animals can indeed ignore speaker-related variation in vowel production in order to categorize vowels, and that they do not require exposure to speaker-related variability in speech (for zebra finches) or non-native vowel categories (for humans) to exhibit these behaviors.

These findings allude to the possibility that a species-shared auditory bias for discarding speaker-related variability to enable vowel categorization may exist. Indeed, an auditory bias for ignoring speaker differences in vowel production may explain why even pre-verbal infants succeed in categorizing vowels with speaker-related variability (Kuhl, 1983). The role of experience with speaker-related variability may be to tune the auditory system to the most relevant acoustic parameters that define phonetic categories. This could at least in part explain why Australian adults with experience normalizing speaker-related variability in their native language showed more robust categorization than birds that had no prior experience with speaker-related variability. In support of this idea, adult human participants were more successful at learning novel speech categories when different speakers were presented within a block of trials as opposed to when different speakers were presented sequentially across blocks of trials (i.e., mixed- and blocked-speaker designs, respectively; Chandrasekaran et al., 2014). Experience with speaker-related variability may also partially explain why monolinguals and multilingual Australian English listeners in our study differed in their speaker/sex normalization abilities, with multilingual human listeners performing more similarly to zebra finches than monolingual human listeners in Experiment 1. Specifically, one reason for the poorer performance by multilinguals in our study could be because many of our bilingual and multilingual speakers had a native language without a vowel contrast similar to Dutch / I/-/*ε*/ (e.g., Arabic). These listeners may have had more problems transferring their initial discrimination of the *FAM* vowels to the *NEW* speaker when compared to Australian English monolinguals who have a similar contrast in their native language, namely / I/-/e/ (and therefore could have had much more experience with speaker-related variability for this vowel contrast, especially during infancy when native vowel categories are formed). This possibility is at the core of models of non-native and second language speech perception (for reviews of these models, see Colatoni et al., 2015b; Escudero et al., 2014), which predict higher discrimination accuracy for a non-native phonemic contrast with a similar counterpart in the listeners’ native language than for a non-native contrast that is not found in the listeners’ native language.

Our results are suggestive the existence for a species-shared auditory bias for discarding speaker-related information, but due to the nature of the experimental design, they do not tell us whether zebra finches will preferentially do so if given equal opportunity to categorize vowels according to either phonetic or indexical information (i.e., vowel category versus speaker identity, respectively). This is because we specifically trained our zebra finches to discriminate between two vowels from a single speaker, but never trained them to discriminate between two different speakers and ignore vowel differences. These results thus motivate important future studies that investigate whether zebra finches are capable of flexibly categorizing vowels along speaker identity as well as phonetic identity, what the underlying mechanism might be, and whether these mechanisms parallel those found in humans. Such experiments could provide crucial evidence that zebra finches do not perceive human speech sounds as unitary wholes (i.e., exemplars), but are able to analytically deconstruct such sounds into different components. Based on visual categorization experiments, it has been suggested that the ability to analyze stimuli that differ along multiple dimensions and categorize them along different dimensions is a recent evolutionary development restricted to primates (Berg and Grace, 2011; Berg et al., 2014; Smith et al., 2011, 2012). As zebra finches are one of the most important models for comparative studies for understanding the evolution of speech perception and language, it is fundamental that we rigorously test whether this assumption is also valid for acoustic categorization (and specifically for human speech sound categorization) in order to thoroughly understand the similarities and differences in how zebra finches perceive speech sounds compared to humans.

The influence of auditory experiences on an auditory bias toward disregarding speaker-information for vowel categorization is further supported by our results showing that not all birds or humans performed equally well when categorizing the novel speaker’s vowels. These individual differences in performance indicate that vowel categorization (that may employ normalization processes) is not uniform across all listeners: not all individuals appear to use the same acoustic cues, or they may use the same cues but to different extents. Heterogenic cue use been reported in voice recognition, where different listeners use different cues to distinguish voices (Kreiman et al., 1992). Linguistic background may have contributed to variation in categorization performance in our study. However, examination of participants with the same linguistic background (i.e., participants 42, 77, and 75) showed that they are not consistent in their errors when classifying the vowels of the unfamiliar speaker (Figures 4 and 5). Consequently, we suspect that an important source of individual variation in vowel categorization may stem from variation in learning strategies used to learn the initial discrimination of the familiar speaker’s vowels (e.g., bird 257; Uno et al., 1997; Morisaka and Okanoya, 2008; van Heijningen et al., 2009, 2013; Seki et al., 2013; Chandrasekaran et al., 2014).

The results of this study provide the first behavioral evidence challenging Monahan and Idsardi’s (2010) hypothesis that normalization of speaker differences in vowel production is achieved by perceptual transformations of auditory input into formant ratios. This hypothesis was based on their finding that age and gender variation contributed significantly to differences in formant frequencies F0 to F3, but that age and gender did not contribute to differences in F1/F3 or F2/F3 ratios. Consequently, we predicted that formant ratios would be used to normalize vowels of speakers of different genders, especially when F1 and F2 values are uninformative of vowel category. Surprisingly, our analyses of humans’ and birds’ performance in such a situation showed that behavioral vowel categorization was not likely achieved by transformation of formants into formant ratios F1/F3 and F2/F3.

Several possibilities can account for the discrepancy between our findings and those of Monahan and Idsardi (2010). For instance, the use of F1/F3 and F2/F3 ratios for speaker normalization may be limited for use with native vowels only, and is only employed after listeners have had exposure to speaker-related variability. This may explain why the brains of monolingual English adult participants were responsive to the F1/F3 ratio of synthesized English vowels (Monahan and Idsardi, 2010), and why Australian English adults do not appear to use formant ratios to categorize Dutch vowels. Of course, it is possible that the brains of our human participants also calculated the F1/F3 ratio, but that this calculation was not used because of interference by processes involved in behavioral responses. Future studies can address these possibilities by recording brain activity for F1/F3 during a behavioral categorization task, using a homogenous linguistic population, artificial speech sounds controlling for various potential confounds, and using very different non-native vowels to avoid perceptual assimilation of non-native vowels into native vowel categories (Best, 1994).

Other normalization methods that involve transformations of formant frequencies into formant ratios—such as Lobanov’s (1971) z-score transformation and Nearey’s (1978) single log mean—may yield better normalization of Dutch vowels, as a comparison of various vowel normalization methods showed that these procedures were the most effective at removing physiological/anatomical variation in Dutch vowels (Adank et al., 2004a). However, these procedures assume that the information required for vowel normalization is distributed across multiple vowel categories (especially /i/, /u/, and /a/) and hence not contained in a single vowel of a speaker. As our subjects heard only / I/ and /*ε*/ vowels of each speaker, it seems unlikely that these normalization procedures would have been used by subjects in this study. Alternatively, perhaps no transformations of the acoustic signal are required for dealing with speaker-related variability. For instance, exemplar-based models of speech perception propose that listeners compare the acoustic properties of the target speech with the acoustic properties of a set of exemplars (gained from past experiences) of that speech category (see Johnson, 1997). However, our results seem to argue against these models as well, since zebra finches in our experiment were able to successfully categorize acoustically similar vowels produced by a novel speaker even though they had prior experience with only one speaker (i.e., they would not have accumulated enough exemplars of each vowel category to enable reliable comparisons). Similar issues apply to computational models of speech perception that use a Bayesian inferential approach—these models must first learn from many speakers/instances in order to be able to categorize novel instances (e.g., Yildiz et al., 2013).

Although an exhaustive exploration of the possible normalization procedures used by the humans and zebra finches in this study is beyond the scope of this paper, we believe it is very important to underscore that a better understanding of how songbirds perceptually compensate for inter-individual variability in their own communication signals may pave the road to a better understanding of how humans compensate for inter-speaker variability in speech. This is because there are many parallels between humans and songbirds in vocal production mechanisms, the acoustic characteristics of vocal output, auditory perception, and the necessity to accurately perceive and categorize vocal communication signals that vary between different individuals.

One example of the striking similarity between how humans produce speech and how birds produce song is the physical mechanisms of phonation. In songbirds, phonation is achieved via a structure called the syrinx, which has two pairs of internal labia that can alter the airflow passing from the lungs to the trachea; specific muscles that control tension of the labia act in coordination with respiratory muscles to generate and supply vocal output with desired acoustic features (Mindlin and Laje, 2005; Riede and Goller, 2010; Amador and Mindlin, 2014). These coordinated motor movements are precisely timed with neural activity in a cortical premotor region required for song perception and production—similarly, human speech requires precise coordination between brain activity and motor control (Amador et al., 2013). Humans and songbirds also seem to share mechanisms of frequency modulation. In humans, subglottal pressure affects fundamental frequency; in zebra finches pressure from air sacs below the syrinx modulates fundamental frequency of vocalizations with harmonic stacks (Amador and Margoliash, 2013). Additionally, songbirds can use their vocal tract as a filter to further acoustically modify the output of the syrinx (for example, by opening and closing of the beak to alter vocal tract length; Nowicki, 1987; Hoese et al., 2000). For zebra finches, Ohms et al. (2010b) showed that both beak gape and the oropharyngeal-esophageal cavity contribute to vocal tract filtering. Similarities in frequency modulation mechanisms between humans and zebra finches may consequently explain why the acoustic structure of human and zebra finch vocal output (i.e., vowels and zebra finch calls) both have broadband harmonic structures with high spectral complexity and low fundamental frequency (Amador and Margoliash, 2013). Zebra finches and humans also share aspects of auditory perception in that both species are sensitive to absolute and relative frequencies (Weisman et al., 1994, 1998, 2004, 2014). As vowel identity is encoded in relative formant frequencies and speaker identity is more closely tied to absolute formant frequencies, zebra finches’ sensitivity to both absolute and relative frequencies may explain why they are able to separate vowel information from speaker information. Lastly, in humans and songbirds, the same vocal mechanism produces both the (linguistic) message and information about the signaler, so that both the message and variation in the message arising from individual differences in the signaler are encrypted in the same signal.

Thus, humans and songbirds face similar challenges in decoding biologically relevant signals (Kriengwatana et al., 2015). How this is done (either through transformations of the acoustic signal, i.e., normalization, or by other types of perceptual learning and categorization principles) remains to be determined. Nonetheless, given the many similarities between vocal production, characteristics of the acoustic vocal signal, auditory perception, and need for accurate perceptual categorization in humans in songbirds, it may not be so surprising that there are also parallels in perceptual mechanisms that allow both species to overcome the problem of separating variability associated with content of the signal from variability arising from the individual signaler. This could potentially explain why zebra finches without experience in categorizing human speech can still successfully partial out speaker-dependent variation and classify vowels based on phonetic information.

In conclusion, our study shows that vowel categorization, despite speaker and gender differences in vowel production, may be achieved without exposure to inter-speaker variability. These results are suggestive of an auditory bias for discounting inter-speaker variability in vowel production that is shared across species and not unique to humans. As current normalization procedures do not sufficiently explain our behavioral results, our findings necessitate novel approaches that can explain how listeners accommodate for speaker differences in vowels without prior experience with different speakers. Incorporation of features or processes that songbirds use to recognize and categorize elements of songs despite inter-individual differences in production may be an important and necessary step to achieving a effective yet parsimonious method of speaker normalization in human speech.

### Conflict of Interest Statement

The authors declare that the research was conducted in the absence of any commercial or financial relationships that could be construed as a potential conflict of interest.

## References

[B1] Adank P. Smits R. van Hout R. (2004a). A comparison of vowel normalization procedures for language variation research. J. Acoust. Soc. Am. 116, 3099–3107. 10.1121/1.1795335 15603155

[B2] Adank P. van Hout R. Smits R. (2004b). An acoustic description of the vowels of Northern and Southern Standard Dutch. J. Acoust. Soc. Am. 116, 1729–1738. 10.1121/1.1779271 15478440

[B3] Amador A. Margoliash D. (2013). A mechanism for frequency modulation in songbirds shared with humans. J. Neurosci. 33, 11136–11144. 10.1523/JNEUROSCI.5906-12.2013 23825417 PMC3718373

[B4] Amador A. Mindlin G. B. (2014). Low dimensional dynamics in birdsong production. Eur. Phys. J. B 87, 300 10.1140/epjb/e2014-50566-5

[B5] Assmann P. F. Nearey T. M. Hogan J. T. (1982). Vowel identification: orthographic, perceptual, and acoustic aspects. J. Acoust. Soc. Am. 71, 975–989. 7085986 10.1121/1.387579

[B6] Amador A. Perl Y. S. Mindlin G. B. Margoliash D. (2013). Elemental gesture dynamics are encoded by song premotor cortical neurons. Nature 495, 59–64. 10.1038/nature11967 23446354 PMC3878432

[B7] Baru A. V. (1975). “Discrimination of synthesized vowels [a] and [i] with varying parameters in dog,” in Auditory Analysis and the Perception of Speech, eds Fant G. Tatham M. A. A. (London: Academic Press), 91–101.

[B8] Berg M.E. Ward M.D. Dai Z. Arantes J. Grace R.C. (2014). Comparing performance of humans and pigeons in rule-based visual categorization tasks. Learn. Mot. 45, 44–58.

[B9] Berg M. E. Grace R. C. (2011). Categorization of multidimensional stimuli by pigeons. J. Exp. Anal. Behav. 95, 305–326. 10.1901/jeab.2010.94-305 21547069 PMC3088496

[B10] Best C. T. (1994). “The emergence of native-language phonological influences in infants: a perceptual assimilation model,” in The Development of Speech Perception: The Transition From Speech Sounds to Spoken Words, eds Goodman J. Nusbaum H. C. (Cambridge: MIT Press), 167–224.

[B11] Bizley J. K. Walker K. M. M. King A. J. Schnupp J. W. H. (2013). Spectral timbre perception in ferrets: discrimination of artificial vowels under different listening conditions. J. Acoust. Soc. Am. 133, 365–376. 10.1121/1.4768798 23297909 PMC3783993

[B12] Boersma P. (2001). Praat, a system for doing phonetics by computer. Glot Int. 5, 341–345.

[B13] Burdick C. K. Miller J. D. (1975). Speech perception by the chinchilla: discrimination of sustained /a/ and /i/. J. Acoust. Soc. Am. 58, 415–427. 1184835 10.1121/1.380686

[B14] Chandrasekaran B. Yi H.-G. Maddox W. T. (2014). Dual-learning systems during speech category learning. Psychon. Bull. Rev. 21, 488–495. 10.3758/s13423-013-0501-5 24002965 PMC3874422

[B15] Chen J. van Rossum D. ten Cate C. (2015). Artificial grammar learning in zebra finches and human adults: XYX versus XXY. Anim. Cogn. 18, 151–164. 10.1007/s10071-014-0786-4 25015135

[B16] Colatoni L. Steele J. Escudero P. (2015a). “Part III. Case studies and analysis of L2 speech perception and production: vowels,” in Second Language Speech Theory and Practice, (Cambridge University Press).

[B17] Colatoni L. Steele J. Escudero P. (2015b). “Theoretical concepts and frameworks,” in Second Language Speech Theory and Practice (Cambridge University Press).

[B18] Creel S. C. Bregman M. R. (2011). How talker identity relates to language processing. Lang. Linguist. Compass 5, 190–204. 10.1111/j.1749-818X.2011.00276.x

[B19] Dewson J. H. (1964). Speech sound discrimination by cats. Science 144, 555–556. 14194105 10.1126/science.144.3618.555

[B20] Dewson J. H. Pribram K. H. Lynch J. C. (1969). Effects of ablations of temporal cortex upon speech sound discrimination in the monkey. Exp. Neurol. 24, 579–591. 4979090 10.1016/0014-4886(69)90159-9

[B21] Dooling R. J. Brown S. D. (1990). Speech perception by budgerigars (*Melopsittacus undulatus*): spoken vowels. Percept. Psychophys. 47, 568–574. 2367177 10.3758/bf03203109

[B22] Edmonds B. A. James R. E. Utev A. Vestergaard M. D. Patterson R. D. Krumbholz K. (2010). Evidence for early specialized processing of speech formant information in anterior and posterior human auditory cortex. Eur. J. Neurosci. 32, 684–692. 10.1111/j.1460-9568.2010.07315.x 20646047

[B23] Eriksson J. L. Villa A. E. P. (2006). Learning of auditory equivalence classes for vowels by rats. Behav. Processes 73, 348–359. 10.1016/j.beproc.2006.08.005 16997507

[B24] Escudero P. Bion R. A. H. (2007). Modeling vowel normalization and sound perception as sequential processes. Proc. Int. Congr. Phonetic Sci. 141, 3–1416.

[B25] Escudero P. Sisinni B. Grimalidi M. (2014). The effect of vowel inventory and acoustic properties in Salento Italian learners of Southern British English vowels. J. Acoust. Soc. Am. 135, 1577–1584. 10.1121/1.4864477 24606292

[B26] Fitch T. W. Giedd J. (1999). Morphology and development of the human vocal tract: a study using magnetic resonance imaging. J. Acoust. Soc. Am. 106, 1511–1522. 10489707 10.1121/1.427148

[B27] Haesler S. Rochefort C. Georgi B. Licznerski P. Osten P. Scharff C. (2007). Incomplete and inaccurate vocal imitation after knockdown of FoxP2 in songbird basal ganglia nucleus area X. PLoS Biol. 5:e321. 10.1371/journal.pbio.0050321 18052609 PMC2100148

[B28] Hillenbrand J. Getty L. A. Clark M. J. Wheeler K. (1995). Acoustic characteristics of American English vowels. J. Acoust. Soc. Am. 97, 3099–3111. 7759650 10.1121/1.411872

[B29] Hoese W. J. Podos J. Boetticher N. C. Nowicki S. (2000). Vocal tract function in birdsong production: experimental manipulation of beak movements. J. Exp. Biol. 203, 1845–1855. 10821742 10.1242/jeb.203.12.1845

[B30] Holt L. L. Lotto A. J. (2010). Speech perception as categorization. Atten. Percept. Psychophys. 72, 1218–1227. 10.3758/APP.72.5.1218 20601702 PMC2921848

[B31] Johnson K. (1997). “Speech perception without speaker normalization: an exemplar model,” in Talker Variability in Speech Processing, eds Johnson K. Mullinex J. (San Diego: Academic Press), 145–165.

[B32] Johnson K. A. (2008). “Speaker Normalization in Speech Perception,” in The Handbook of Speech Perception, eds Pisoni D. B. Remez R. E. (Oxford: Blackwell), 363–389. 10.1002/9780470757024.ch15

[B33] Kreiman J. Gerratt B. R. Precoda K. Berke G. S. (1992). Individual differences in voice quality perception. J. Speech Hear Res. 35, 512–520. 1608242 10.1044/jshr.3503.512

[B34] Kriengwatana B. Escudero P. ten Cate C. (2015). Revisiting vocal perception in non-human animals: a review of vowel discrimination, speaker voice recognition, and speaker normalization. Front. Psychol. 5:1543. 10.3389/fpsyg.2014.01543 25628583 PMC4292401

[B35] Kuhl P. K. (1983). Perception of auditory equivalence classes for speech in early infancy. Infant Behav. Dev. 6, 263–285. 10.1016/S0163-6383(83)80036-8

[B36] Lobanov B. M. (1971). Classification of Russian vowels spoken by different speakers. J. Acoust. Soc. Am. 49, 606–608.

[B37] Magnuson J. S. Nusbaum H. C. (2007). Acoustic differences, listener expectations, and the perceptual accommodation of talker variability. J. Exp. Psychol. Hum. Percept. Perform. 33, 391–409. 10.1037/0096-1523.33.2.391 17469975

[B38] Miller J. D. (1989). Auditory-perceptual interpretation of the vowel. J. Acoust. Soc. Am. 85, 2114–2134. 10.1121/1.397862 2659639

[B39] Mindlin G. B. Laje R. (2005). The Physics of Birdsong. Berlin: Springer Verlag.

[B40] Monahan P. J. Idsardi W. J. (2010). Auditory sensitivity to formant ratios: toward an account of vowel normalization. Lang. Cogn. Process. 25, 808–839. 10.1080/01690965.2010.490047 20606713 PMC2893733

[B41] Mooney R. (2009). Neural mechanisms for learned birdsong. Learn. Mem. 16, 655–669. 10.1101/lm.1065209 19850665

[B42] Morisaka T. Okanoya K. (2008). Cognitive tactics of Bengalese finch (*Lonchura striata* var. domestica) for song discrimination in a go/no-go operant task. J. Ethol. 27, 11–18. 10.1007/s10164-007-0074-8

[B43] Nearey T. M. (1978). Phonetic Feature Systems for Vowels. Indiana: Indiana University Linguistics Club.

[B44] Nowicki S. (1987). Vocal tract resonances in oscine bird sound production: evidence from birdsongs in a helium atmosphere. Nature 325, 53–55. 10.1038/325053a0 3796738

[B45] Ohms V. R. Escudero P. Lammers K. ten Cate C. (2012). Zebra finches and Dutch adults exhibit the same cue weighting bias in vowel perception. Anim. Cogn. 15, 155–161. 10.1007/s10071-011-0441-2 21761144 PMC3281197

[B46] Ohms V. R. Gill A. Van Heijningen C. A. A. Beckers G. J. L. ten Cate C. (2010a). Zebra finches exhibit speaker-independent phonetic perception of human speech. Proc. R. Soc. B 277, 1003–1009. 10.1098/rspb.2009.1788 19955157 PMC2842761

[B47] Ohms V. R. Snelderwaard P. C. ten Cate C. Beckers G. J. L. (2010b). Vocal tract articulation in zebra finches. PLoS ONE 5:e11923. 10.1371/journal.pone.0011923 20689831 PMC2912855

[B48] Peterson G. E. Barney H. H. (1952). Control methods used in a study of the vowels. J. Acoust. Soc. Am. 24, 175–184.

[B49] Potter R. K. Steinberg J. C. (1950). Toward the specification of speech. J. Acoust. Soc. Am. 22, 807–820.

[B50] Riede T. Goller F. (2010). Peripheral mechanisms for vocal production in birds–differences and similarities to human speech and singing. Brain Lang. 115, 69–80. 10.1016/j.bandl.2009.11.003 20153887 PMC2896990

[B51] Seki Y. Suzuki K. Osawa A. M. Okanoya K. (2013). Songbirds and humans apply different strategies in a sound sequence discrimination task. Front. Psychol. 4:447. 10.3389/fpsyg.2013.00447 23882247 PMC3713397

[B52] Smith J. D. Ashby F. G. Berg M. E. Murphy M. S. Spiering B. Cook R. G. (2011). Pigeons’ categorization may be exclusively nonanalytic. Psychon. Bull. Rev. 18, 414–421. 10.3758/s13423-010-0047-8 21327382 PMC3532937

[B53] Smith J. D. Berg M. E. Cook R. G. Murphy M. S. Crossley M. J. Boomer J. (2012). Implicit and explicit categorization: a tale of four species. Neurosci. Biobehav. Rev. 36, 2355–2369. 10.1016/j.neubiorev.2012.09.003 22981878 PMC3777558

[B54] Spierings M. J. ten Cate C. (2014). Zebra finches are sensitive to prosodic features of human speech. Proc. R. Soc. B 281, 20140480. 10.1098/rspb.2014.0480 24870039 PMC4071541

[B55] Strange W. Verbrugge R. R. Shankweiler D. P. Edman T. R. (1976). Consonant environment specifies vowel identity. J. Acoust. Soc. Am. 60, 213–224. 956528 10.1121/1.381066

[B56] Stroup W. (2012). Generalized Linear Mixed Models: Modern Concepts, Methods and Applications. Boca Raton: CRC Press,

[B57] Syrdal A. K. Gopal H. S. (1986). A perceptual model of vowel recognition based on the auditory representation of American English vowels frequency. J. Acoust. Soc. Am. 79, 1086–1100. 3700864 10.1121/1.393381

[B58] Tuomainen J. Savela J. Obleser J. Aaltonen O. (2013). Attention modulates the use of spectral attributes in vowel discrimination: behavioral and event-related potential evidence. Brain Res. 1490, 170–183. 10.1016/j.brainres.2012.10.067 23174416

[B59] Uno H. Maekawa M. Kaneko H. (1997). Strategies for harmonic structure discrimination by zebra finches. Behav. Brain Res. 89, 225–228. 9475629 10.1016/s0166-4328(97)00064-8

[B60] van Heijningen C. A. A. de Visser J. Zuidema W. ten Cate C. (2009). Simple rules can explain discrimination of putative recursive syntactic structures by a songbird species. Proc. Natl. Acad. Sci. U.S.A. 106, 20538–20543. 10.1073/pnas.0908113106 19918074 PMC2787117

[B61] van Heijningen C. A. A. Chen J. van Laatum I. van der Hulst B. ten Cate C. (2013). Rule learning by zebra finches in an artificial grammar learning task: which rule? Anim. Cogn. 16, 165–175. 10.1007/s10071-012-0559-x 22971840

[B62] von Kriegstein K. Warren J. D. Ives D. T. Patterson R. D. Griffiths T. D. (2006). Processing the acoustic effect of size in speech sounds. Neuroimage 32, 368–375. 10.1016/j.neuroimage.2006.02.045 16644240

[B63] Weisman R. G. Njegovan M. G. Williams M. T. Cohen J. S. Sturdy C. B. (2004). A behavior analysis of absolute pitch: sex, experience, and species. Behav. Processes 66, 289–307. 10.1016/j.beproc.2004.03.010 15157978

[B64] Weisman R. Hoeschele M. Sturdy C. B. (2014). A comparative analysis of auditory perception in humans and songbirds: a modular approach. Behav. Processes 104, 35–43. 10.1016/j.beproc.2014.02.006 24565980

[B65] Weisman R. Njegovan M. Ito S. (1994). Frequency ratio discrimination by zebra finches (*Taeniopygia guttata*) and Humans (*Homo sapiens*). J. Comp. Psychol. 108, 363–372. 10.1037/0735-7036.112.3.244 9770314

[B66] Weisman R. Njegovan M. Sturdy C. Phillmore L. Coyle J. Mewhort D. (1998). Frequency-range discriminations: special and general abilities in zebra finches (*Taeniopygia guttata*) and Humans (*Homo sapiens*). J. Comp. Psychol. 112, 244–258. 9770314 10.1037/0735-7036.112.3.244

[B67] Yildiz I. B. von Kriegstein K. Kiebel S. J. (2013). From birdsong to human speech recognition: bayesian inference on a hierarchy of nonlinear dynamical systems. PLoS Comput. Biol. 9:e1003219. 10.1371/journal.pcbi.1003219 24068902 PMC3772045

